# Human papillomavirus in premalignant oral lesions: No evidence of association in a Spanish cohort

**DOI:** 10.1371/journal.pone.0210070

**Published:** 2019-01-16

**Authors:** Sara Gomez-Armayones, Eduardo Chimenos-Küstner, Antonio Marí, Sara Tous, Rosa Penin, Omar Clavero, Beatriz Quirós, Miguel Angel Pavon, Miren Taberna, Laia Alemany, Octavio Servitje, Marisa Mena

**Affiliations:** 1 Department of Dermatology, Bellvitge University Hospital, L’Hospitalet de Llobregat, Barcelona, Spain; 2 Department of Medicine, Bellvitge University Hospital, L’Hospitalet de Llobregat, Barcelona, Spain; 3 Department of Odontostomatology, Odontological University Hospital of Barcelona, L’Hospitalet de Llobregat, Barcelona, Spain; 4 Department Oral and Maxillofacial Surgery, Bellvitge University Hospital, L’Hospitalet de Llobregat, Barcelona, Spain; 5 Cancer Epidemiology Research Program, IDIBELL, Catalan Institute of Oncology (ICO), L’Hospitalet de Llobregat, Barcelona, Spain; 6 CIBER in primary and secondary prevention of viral induced cancers (CIBERONC), Madrid, Spain; 7 Department of Pathology, Bellvitge University Hospital, L’Hospitalet de Llobregat, Barcelona, Spain; 8 Department of Medical Oncology, Catalan Institute of Oncology (ICO), ONCOBELL, IDIBELL, L’Hospitalet de Llobregat, Barcelona, Spain; 9 University of Barcelona, Barcelona, Spain; 10 Epidemiology and Public Health, Centro de Investigación Biomédica en Red: Epidemiología y Salud Pública (CIBERESP), Instituto de Salud Carlos III, Madrid, Spain; Istituto Nazionale Tumori IRCCS Fondazione Pascale, ITALY

## Abstract

**Background:**

Human papillomavirus (HPV) is the cause of a fraction of head and neck squamous cell carcinoma. Although this relation is well-known, it is still not clear the role of HPV in premalignant oral lesions such as oral lichen planus (OLP) and dysplasia. We aimed to evaluate the HPV-DNA prevalence and type distribution in a set of oral biopsies obtained from patients diagnosed with OLP and dysplasia, as well as the role of HPV in these lesions.

**Methods:**

A retrospective cohort of all premalignant oral lesions consecutively diagnosed from March 30^th^ 1995 to May 21^st^ 2014 at Hospital of Bellvitge and Odontological University Hospital of Bellvitge was identified and classified in four groups: OLP (groups 1 and 2) and dysplasias (groups 3 and 4) that progressed or not to invasive cancer during follow-up. A random selection targeting 25 cases was aimed to be performed for each group. All selected cases were subjected to pathological evaluation, DNA quality control and HPV-DNA detection. HPV-DNA positive samples were further subject to p16^INK4a^ analysis.

**Results:**

A total of 83 cases yielded a valid HPV-DNA result. From those, 7 and 34 cases were OLP that progressed or not to invasive cancer during follow-up, whereas 24 and 18 cases were displasias that progressed or not to invasive cancer during follow-up, respectively. HPV-DNA was detected in 4 samples (3 dysplastic lesions and 1 OLP). Two samples were HPV16 positive (2%), 1 sample HPV18 positive (1%) and 1 sample (1%) was HPV indeterminate. Two out of four HPV-DNA positive cases had high p16^INK4a^ expression and none of the HPV positive cases progressed to invasive cancer during long-term follow-up.

**Conclusions:**

We found a low HPV-DNA attributable fraction in premalignant lesions of the oral cavity, suggesting that HPV is unlikely to play a significant role in oral carcinogenesis in our setting.

## Introduction

Oral squamous cell carcinoma (OSCC) is the most common type of head and neck squamous cell carcinoma (HNSCC), with an estimated 200,000 new cases every year [1]. OSCC may be preceded by premalignant oral lesions, including mucosal lesions such as oral lichen planus (OLP), leukoplakia, erythroplakia or more widespread conditions, some of them showing different grades of dysplasia at histopathological analysis [2].

HPV attributable fractions in OSCC are estimated at 2.2%, substantially lower than those for oropharyngeal squamous cell carcinoma, estimated a 30,8% [1]. The relationship between HPV infection and premalignant oral lesions is still controversial [2,3], as it is the identification of HPV-related precursor lesions in the head and neck [4]. Some authors pointed out that there are huge geographic differences in prevalence of HPV infection in oral mucosal disorders worldwide [5], which may be partially explained by differences in case selection, risk factors, ethnicity, sampling or detection tecniques [6,7].

Premalignant oral lesions include oral leucoplakia, oral erythroplakia, oral proliferative verrucous leukoplakia, oral submucous fibrosis, oral lichen planus (OLP) and actinic cheilitis [8,9]. In our study we evaluated OLP because it is the most common premalignant condition, as well as dysplasia, because it is considered a previous stage of progression to invasive cancer [10]. OLP is a T-cell-mediated chronic inflammatory disease [11] that affects around 1% of population. Its ethiopathogenesis is poorly understood [8]. Histologically, lesions are characterized by hyperkeratosis, basal layer liquefaction of the oral epithelium-connective tissue interface and a dense infiltration of a band of lymphocytes [12]. Some studies show that HPV may play a role in OLP genesis [13,14], but others disagree [15,16]. High-risk HPV16 and HPV18 are the most prevalent types found in OLP [14,15,17] and its eventual malignant transformation [18]. However, contrary to the OLP subtype, as well as other factors as smoking and buccal hygiene, the role of high-risk HPV in progression to invasive cancer has not been well established [19,20,21].

Dysplasia is also considered a potentially malignant disorder, as some studies relate oral dysplasia with HNSCC [22,23]. It is considered a clinical as well as a pathological concept, as some lesions such as leucoplakia may show dysplasia at the pathological study [24]. The proportion of HPV-DNA positive cases in dysplasia have found to be heterogeneous (between 6% to 27.1%) [20,25,26]. Again, the role of HPV in the development of oral dysplasia and its subsequent malignancy is not still clear as most of the studies and meta-analyses show discrepant results [21,25].

We aimed to assess the prevalence of HPV infection in a Spanish series of OLP and dysplasia, the type distribution of HPV-DNA positive cases and the role of the virus in these lesions, evaluating if they expressed p16^INK4a^ and progressed to invasive cancer during follow-up or not.

## Materials and methods

### Study design and samples

A retrospective cohort study of all patients consecutively diagnosed from March 30^th^ 1995 to May 21^st^ 2014 with OLP and oral dysplasia at Bellvitge University Hospital and Odontological University Hospital of Barcelona (Spain) with available clinical data was conducted. All selected patients had to fulfil pre-established inclusion criteria: to be diagnosed with OLP or oral dysplasia in the pre-established anatomical locations (base of the tongue, tongue, oropharynx, gingiva, floor of the mouth, palate, cheek mucosa and oral cavity not specified) and not having had a previous oral cavity cancer or oropharyngeal cancer. Since the causal role of HPV in HNSCC is only consistently established in oropharyngeal cancer and moreover, the identification of HPV-related precursor lesions in the head and neck remains puzzling [4], we considered pertinent to keep those premalignant lesions in the study. External lip lesions were not included in the study as they are not considered to be related to HPVs [23]. Data was retrieved on demographics, smoking and alcohol consumption, clinical and follow-up data. We classified the whole cohort (n = 421) in four groups: OLP (groups 1 and 2) and dysplasias (groups 3 and 4) that progressed or not to invasive cancer during follow-up, see Table 1. Once the classification was made, we aimed to test in a first stage a pilot group of 100 cases since we expected HPV prevalence to be very low. Depending on the observed HPV prevalence, we would decide to test or not the whole cohort in a second stage. In order to select the pilot group of 100 cases, we planned to perform a random selection of 25 cases for each group (i.e. groups 1 to 4). However, there were not 25 but only 7 OLP with available paraffin block that progressed to invasive cancer during follow up cases in the whole cohort (n = 421). Thus, all those 7 cases were included. In order to still testing 50 OLP in the pilot, sample was then enriched up to 43 randomly selected OLP that not progressed to invasive cancer during follow up.

**Table 1:** Type of lesion and progression to invasive cancer.

Type of lesion	Group	Total N	N pilot^a^
**OPL**			
*Did progress to invasive cancer during follow up*?			
YES	1	25^b^	7
NO	2	289	34
**Dysplasia**			
*Did progress to invasive cancer during follow up*?			
YES	3	55	24
NO	4	52	18
**Total**	-	421	102^c^

^a^In order to select the pilot cases for each group of lesions to be tested for HPV-DNA, a random selection of 25 cases was made when possible (i.e. more than 25 cases with available sample in the whole cohort (n = 421) for the specific group).

^b^Only 7 out of 25 with available sample.

^c^The selected 102 cases were further evaluated when arrived at ICO as described in the materials and methods section and finally 83 cases were tested for HPV-DNA (see Fig 1).

OLP: Oral lichen planus

It was requested to provide samples using a common protocol from sample selection, retrieval, processing and shipping to Catalan Institute of Oncology (ICO) [27].

### Pathology and laboratory procedures

Pathology procedures were performed at Hospital of Bellvitge and ICO and laboratory procedures at the ICO according to a robust and well validated methodology [27–29]. Before being shipped to ICO, all paraffin embedded samples were revised by the pathologist at origin to confirm diagnose. Blocks were re-embedded whenever necessary and four paraffin sections were obtained for each block. In order to assess potential carryover contamination at the local level, we additionally requested tissue samples of patients with non-HPV related diagnoses processed in the same laboratory and close to the patients’ diagnosis time. Blocks were processed under strict conditions to avoid DNA contamination [27–29]. First and last sections were stained with haematoxylin and eosin (H&E) for pathological revaluation, which included confirmation of OLP and dysplasia. A histopathological form was developed for the study by two pathologists (see S1 Fig).

A sample was determined to be adequate for HPV testing if the lesion of interest was observed in both H&E-stained sections of the study specimen. Intermediate sections were used for HPV-DNA detection and genotyping and p16^INK4a^ immunohistochemistry (IHC) [30]. For HPV-DNA detection and genotyping, samples were first treated with 250 μl of freshly prepared proteinase K solution to extract DNA. Short polymerase chain reaction (PCR) fragment using biotin labelled SPF10 primers was performed using 10 μl of a 1:10 dilution of the crude DNA isolate in a final reaction volume of 50 μl. The amplified PCR products were tested using a probe hybridization step with a cocktail of conservative probes recognizing at least 54 mucosal HPV genotypes for the detection of HPV DNA with a DNA enzyme immunoassay (DEIA). Optical densities (OD450) were read on a microtitre plate reader and categorized as HPV-DNA-negative, -positive, or borderline. Borderline samples were run on the DEIA system again. The amplimers of the DEIA HPV-DNA-positive samples would have been subsequently analysed by reverse hybridization line probe assay (LiPA25) (version 1; Laboratory Biomedical Products, Rijswijk, the Netherlands). The technique can detect 25 high-risk and low-risk HPV types (type numbers 6, 11, 16, 18, 31, 33, 34, 35, 39, 40, 42, 43, 44, 45, 51, 52, 53, 54, 56, 58, 59, 66, 68, 70, 74). In order to evaluate DNA quality, the samples were subjected to a PCR targeting the human tubulin gene, which generated a 65 bp amplicon, the same size as the SPF10 amplicon used for assessing the presence of HPV-DNA. p16^INK4a^ expression was evaluated on HPV-DNA positive cases using the CINtec histology kit (clone E6H4, Roche mtm laboratories AG, Germany), following the manufacturer’s protocol [27–29]. It is generally accepted that p16^INK4a^ staining has a high predictive value to identify HPV-related cancers when the pattern shows a strong and diffuse nuclear and cytoplasmic staining in at least 70% or more of the tumour [31]. However, for our study in premalignant oral lesions we chosen as previously [27] the methodology developed by Halec and colleagues [32] where the cut-off for p16 positivity is a staining greater than 25% in a diffuse or continuous pattern, since we have observed [27] that using p16^INK4a^ and/or HPV-mRNA in addition to HPV-DNA yielded the most accurate approximation to judge HPV carcinogenicity in HNSCC also in non-oropharyngeal sites.

### Ethics

This study had formal approval by the Ethical Committee for Clinical and Epidemiological research of our research centre, Hospital of Bellvitge, Catalan Institute of Oncology (ICO), Odontological University Hospital of Barcelona and Hospital of University of Barcelona (*Comitè Ètic d’Investigació Clínica de l’Hospital Universitari de Bellvitge*, L’Hospitalet de Llobregat, Spain). Study implied the use of archival material only, and it did not envisage any contact with the patients. Adequate measures to ensure data protection, confidentiality, patients’ privacy and anonymization were taken into account. No informed consent was available due to the retrospective design of the study and the large proportion of deceased and untraceable patients. However, informed consent was obtained for a single patient diagnosed in 2014 (n = 1).

## Results

**Fig 1:**
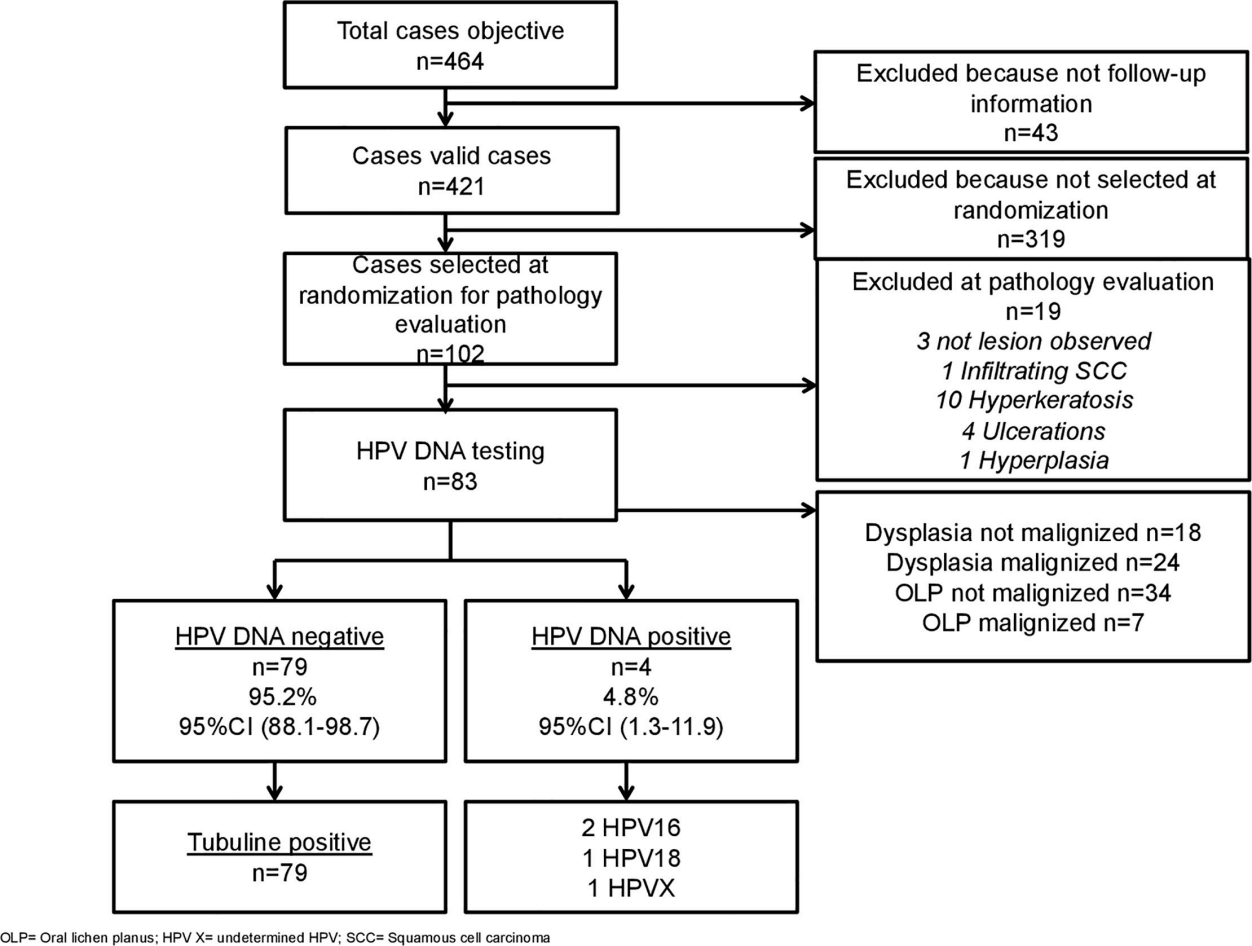
Algorithm.

Fig 1 describes the workflow of the targeted cases, samples collected, processed and finally tested for HPV-DNA. A total of 464 OPL and dysplasia cases were consecutively diagnosed at the Pathology Department of Hospital of Bellvitge and Odontological University Hospital of Barcelona from March 30^th^ 1995 to May 21^st^ 2014. Among these, 43 samples were excluded because follow-up information was not available. From the resulting 421 cases a total of 102 cases were selected for the pilot study on HPV-DNA assessment as previously described in material and methods. While a random selection of 25 cases was possible to be performed for groups 2, 3, 4, only 7 OLP with available paraffin block from the whole cohort progressed to invasive cancer during follow up (see Table 1). Thus, no random selection was possible to be performed in this case and all 7 cases were included in the pilot study. In order to still testing 50 OLP in the pilot, sample was then enriched up to 43 randomly selected OLP that not progressed to invasive cancer during follow up.

After pathology evaluation, 19 samples were excluded (3 with no lesion observed, 1 infiltrating squamous cell carcinoma, 10 hyperkeratosis, 4 ulcerations and 1 hyperplasia). The final number of cases for each group was: 34 OLP that did not progressed to invasive cancer during follow up (group 1), 18 dysplasias that did not progressed to invasive cancer during follow up (group 2), 24 dysplasias that progressed to invasive cancer during follow up (group 3) and 7 OLP that progressed to invasive cancer during follow up (group 4). Two samples from group 2 required a second testing for technique problems and were finally included. All controls were HPV-negative and all HPV-DNA negative cases were tubulin positive.

Characteristics of the samples are presented in Table 2. Mean age was 60.8 (Standard Deviation SD 11.6) at the moment of diagnosis. Forty-eight (57.8%) cases were male and most of the patients (36.1%) were diagnosed between 2010 and 2014. More than half of the patients (45, 54.2%) belonged to non-smoker group while 58 (69.9%) belonged to non-drinker group. Cheek mucosa was the most common anatomical location of the lesions (42.2%). Only 7 patients (8.4%) were diagnosed with a previous neoplasia in other locations than head and neck area. Regarding to pathology diagnosis, 45 (54.2%) lesions were OLP, whereas 13 (15.7%) showed low dysplasia, 12 (14.5%) moderate dysplasia and 13 (15.7%) severe dysplasia. Most cases were treated with surgery (46.8%).

**Table 2:** Demographics and clinical characteristics of patients with oral premalignant lesions.

Characteristics	OPL^a^ samples (n = 83) No. (%)
**Age at diagnosis**. Mean (SD^b^)	60.8 (11.6)
**Gender**	
Male	48 (57.8)
Female	35 (42.2)
**Period of diagnosis**	
1995–1999	6 (7.2)
2000–2004	14 (16.9)
2005–2009	29 (34.9)
2010–2014	34(36.1)
**Tobacco use**	
Non smoker	45 (54.2)
< 20 cigarettes/day	30 (36.1)
≥ 20 cigarettes/day	8 (9.6)
**Alcohol consumption**	
Non drinker	58 (69.9)
< 100 grams/day	23 (27.7)
≥ 100 grams/day	2 (2.4)
**Subsite**	
Gum	10 (12.0)
Cheek mucosa	35 (42.2)
Mobile tongue	22 (26.5)
Floor of the mouth	3 (3.6)
Oral cavity non-specified	9 (10.8)
Oropharynx	4 (4.8)
**Previous neoplasia**	
Yes	7 (8.4)
No	76 (91.6)
**Diagnostic**	
Oral lichen planus	45 (54.2)
Dysplasia low grade	13 (15.7)
Dysplasia moderate grade	12 (14.5)
Dysplasia severe grade	13 (15.7)
**Treatment by diagnostic (n = 79)**	
Oral lichen planus	45 (54.2)
No treatment (clinical control)	13
Topical treatment	14
Surgery	14
CO2 laser	0
Combined treatment	1
Missing	3
Dysplasia low grade	13 (15.7)
No treatment (clinical control)	4
Topical treatment	1
Surgery	5
CO2 laser	1
Oral retinoid	0
Combined treatment	2
Dysplasia moderate grade	12 (14.5)
No treatment (clinical control)	3
Topical treatment	1
Surgery	8
CO2 laser	0
Oral retinoid	0
Dysplasia severe grade	13 (15.7)
No treatment (clinical control)	2
Topical treatment	0
Surgery	9
CO2 laser	0
Oral retinoid	0
Combined treatment	1
Missing	1

^a^OPL: Oral pre-malignant lesions.

^b^SD: Standard deviation.

**Fig 2:**
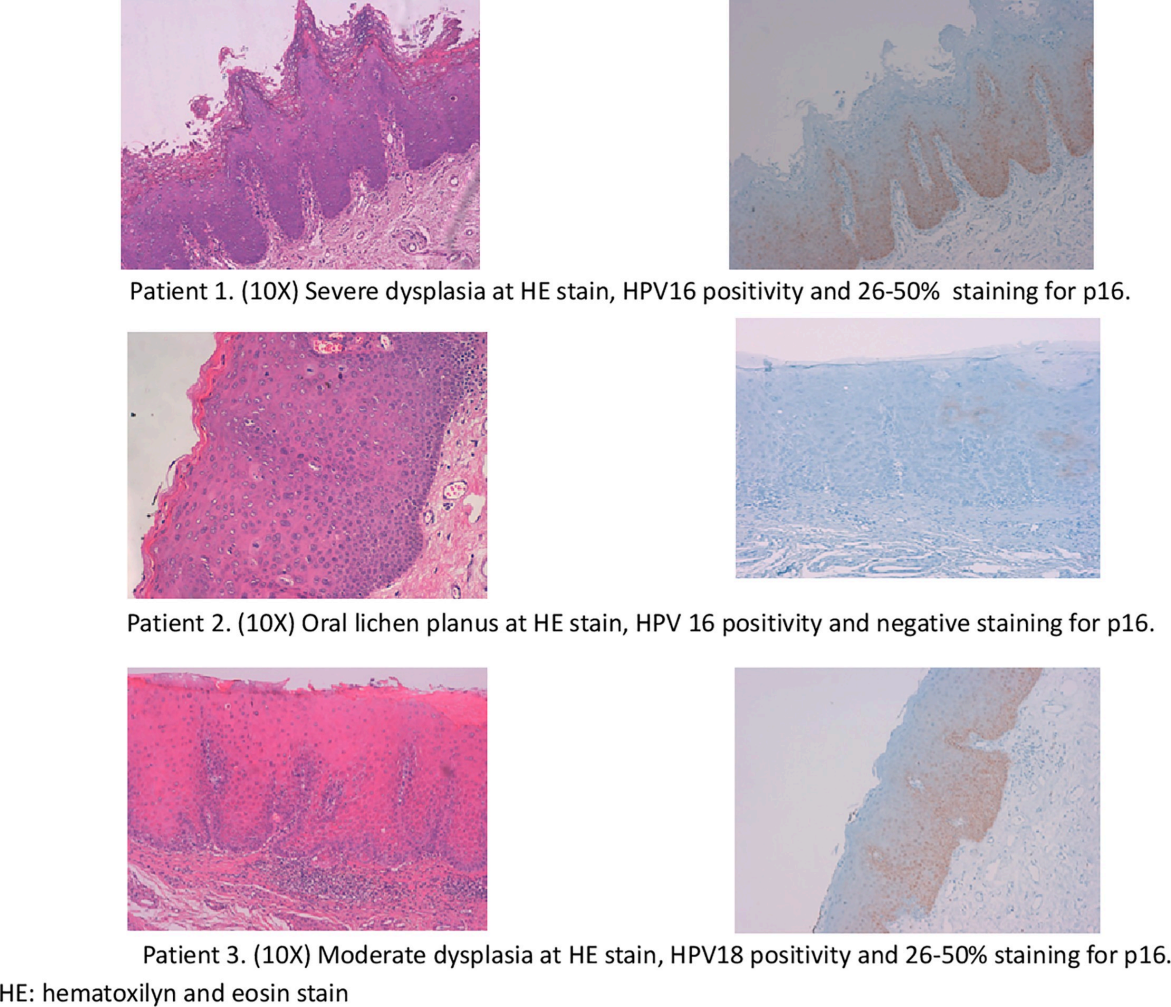
Pictures of HE and p16^INK4a^ IHC of 3 out 4 HPV-DNA positive cases. Patient 1. (10X) Severe dysplasia at HE stain, HPV16 positivity and 26–50% staining for p16. Patient 2. (10X) Oral lichen planus at HE stain, HPV 16 positivity and negative staining for p16. Patient 3. (10X) Moderate dysplasia at HE stain, HPV18 positivity and 26–50% staining for p16. HE: hematoxilyn and eosin stain.

Only four samples were HPV-DNA positive. HPV16 was found in a OLP and in a dysplasia that did not progressed to invasive cancer during follow up, respectively. HPV18 and undetermined HPV were found in severe and low dysplasia that did not progressed to invasive cancer during follow up, respectively. Two out of four HPV-DNA positive cases, which were as severe dysplasia positive for HPV16 and a moderate dysplasia positive for HPV18, showed diffuse expression of p16^INK4a^ with 25 to 50% of cells stained (Fig 2, patient 1 and 3). Both cases showed a nuclear basal and ascendant stain.

One HPV16 positive-case was a 33-year-old male (Fig 2, patient 1), smoker and ex-drinker, HIV-carrier (human immunodeficiency virus) with many comorbidities, who had and unspecific oral lesion at cheek mucosa diagnosed as severe dysplasia with positive p16^INK4a^ stain. Second HPV16 positive sample (patient 2) was a 60-year-old woman, non-smoker and non-alcoholic, with no comorbidities, who developed an OLP in the gum that did not degenerate. The HPV18 positive-case (patient 3) was a 55-year-old smoker and drinker male with many comorbidities who had a moderate dysplasia in a leucoplakia at floor of the mouth with positive p16^INK4a^ stain that healed during follow-up. The undetermined HPV case was a 67-year-old woman, non-smoker and non-alcoholic, with comorbidities presenting a leukoerythroplakia lesion in the tongue diagnosed as low-grade dysplasia that resolved within the next 4 years.

## Discussion

To our knowledge, this is one of the largest studies about HPV and oral premalignant lesions tested in a standardized manner with international, robust and well-validated methodology [27–29]. In Spain, there was only a previous study having tested 35 leukoplakias for HPV-DNA [33].

Association between HPV and OLP and dysplasia has been suggested but it has not been well established. In our study, most of the cases were found at oral cavity and the virus was detected only in 4 cases (4.8%). When examining the positivity by type of lesion, we found one OPL that did not progressed to invasive cancer during follow up (2.9% of all OLP) and three dysplasias that did not progressed to invasive cancer during follow up (7.1% of all dysplasias). This low prevalence limited our ability to analyse factors associated with HPV infection. Likewise, HPV-DNA has been observed in a small proportion (6.4%) of dysplasias in preliminary results of a recent Canadian study [20]. Regarding OLP, a low HPV-DNA prevalence was reported in thirty-seven fresh-frozen tissue biopsy specimens from OLP lesions tested for HPV-DNA where only one sample was positive [34]. Other studies [35,36] also showed very low prevalence in OLP and premalignant conditions. On the other hand, a previous systematic review [25] show a statistically significant twofold difference (23% versus 11%) between HPV associated with OLP lesions and HPV found in normal tissue. However, there is a high degree of variation between studies in experimental methods, selection criteria and specimen collection [37].

Noteworthy, the attributable fraction of HPV in oral cavity cancer has been estimated between 2.2% [1] and 4.4% [27] and the oral prevalence in healthy population at 4.5% [38].

None of our 4 HPV DNA positive cases progressed to invasive cancer during follow-up. To date, there is no clear understanding about HPV natural history at premalignant oral lesions (latency, reactivation or subclinical infection) and it seems that its course depends on the HPV serotype and other associated factors such as immune status, smoking, alcohol, environmental factors, poor oral hygiene and eating habits [23].

Only two out of four HPV-DNA positive cases, which were positive for HPV16 and HPV18, showed a diffuse p16^INK4a^ pattern, representing a 2.4% of the total sample. Preliminary results from the Canadian study showed a HPV-DNA positivity of 6.2% of the dysplasias, whereas 5.5% of all analysable cases demonstrated a p16^INK4a^ diffuse pattern [20]. The study also found that patients with oral dysplasia and focal p16^INK4a^ expression had a significantly lower risk of lesion progression, whereas a diffuse p16^INK4a^ expression did not represent a specific biomarker of HPV-related precancerous lesions in the oral cavity [39]. Results from previous studies are summarized in S1 Table.

Our study has several strengths comprising the combined use of well-validated protocols with highly sensitive assays for HPV-DNA detection and a marker of HPV related transforming process such as p16^INK4a^ expression at positive cases, as it is known that the mere detection of HPV-DNA is not sufficient to establish causality in premalignant oral lesions and HNSCCs [31]. Additionally, we evaluated the quality of our specimens by means of cellular tubulin detection, which was positive in all cases. However, the study also has several limitations. The HPV-positive cases were not tested for HPV E6/E7 mRNA expression, which is considered the gold-standard for classification of an HPV-caused cancer. Moreover, the significance of p16-positivity in the oral cavity is debated [40,41]. However, our previous results from the ICO study [27], where the cut-off for p16 positivity in HNSCCs was a staining greater than 25%, showed that using p16^INK4a^ and/or HPV-mRNA in addition to HPV-DNA yielded the most accurate approximation to judge HPV carcinogenicity also in non-oropharyngeal sites. We tested a small number of samples and we obtained low rates of HPV positivity. However, our population represents a specific geographic region, and our results are consistent with other studies performed in the same area. The low rate of HPV-positivity also deterred us to evaluate HPV infection as prognostic marker. The study was not population based, and thus, it is not possible to exclude some degree of referral or selection bias. For this reason, we tried to minimize this limitation selecting all cases consecutively. We neither have information about the size of the oral lesions in most of the cases nor specific protocol about the treatment of premalignant oral lesions.

After studying a cohort of patients with premalignant oral lesions using a highly sensitive PCR technique for paraffin-embedded tissues, we found a low HPV-DNA attributable fraction in premalignant lesions of the oral cavity, suggesting that HPV is unlikely to play a significant role in oral carcinogenesis in our setting.

## Supporting information

S1 Fig Pathological form. (DOCX)

S1 Table Results from previous studies. (DOCX)
