# Supplementary material for: Human papillomavirus in premalignant oral lesions: No evidence of association in a Spanish cohort
Source: PLoS One. 2019 Jan 16;14(1):e0210070. doi: 10.1371/journal.pone.0210070 (PMC6335078; doi:10.1371/journal.pone.0210070)
Supplement: S1 Fig — (DOCX) [file pone.0210070.s001.docx]

**S1 Fig. Pathological form**

*Identification number _____ Date _____*

*Pathologist ________________*

*Localization*

- Base of the tongue (oropharynx) (C01.9)
- Other regions of oral cavity (oral cavity) (C02.0-3)
- Lingual tonsil (oropharynx) (C02.4)
- Tongue not specified (C02.8-9)
- Gum (C03)
- Floor of the tongue (C04)
- Hard palate (C05.0)
- Soft palate (oropharynx) ( C05.1)
- Uvula (oropharynx) (C05.2)
- Palate not specified (C05.8-9)
- Other region of oral cavity (cheek mucosa, retromolar) (C06.0-8)
- Oral cavity not specified (C06.9)
- Amygdala (C09)
- Oropharynx not tonsillar area not specified
- Other regions of oropharynx (vallecula, posterior wall) (C10.0-4)
- Oropharynx not specified (C10.8-9)
- Lip (mucosal region)
- Others
- Not present

1. **Pathological diagnosis of the original lesion**
   1. Oral lichen planus
   2. Leucoplakia
   3. Ulceration
   4. Epidermisation
   5. Descriptive
   6. Others
2. **Pathological description**
   1. **Epithelial alterations**
      - Atrophy
      - Epithelial hyperplasia
      - Hyperkeratosis / parakeratosis / hypergranulosis
      - Epidermization
      - Ulcer
      - Papillomatous lesion
      - Dysplasia (low / mild / severe / unspecific)
      - Squamous cell carcinoma *in situ*
      - Others __________
   2. **Inflammatory infiltrate**
      - No
      - Yes
        - Lymphocytic / lymphohistiocytic
   - Lichenoid
   - In band
   - Interphase / focal / diffuse
   - Histiocytic
   - Plasmacytic
   - Others __________
   - Grade
     - Low / mild / severe
   1. **Other findings** __________
   2. **Definitive diagnosis of the lesion**
      - Oral lichen planus
      - Mucositis / lichenoid stomatitis
      - Leucoplakia
      - Ulceration
      - Epidermization
      - Epithelial dysplasia with lichenoid infiltrate
      - low / mild / severe / unspecific
      - Dysplasia
      - low / mild / severe / unspecific
      - Squamous cell carcinoma in situ
      - Descriptive __________
3. **Slice A&B**
   - - Same
     - Different
4. **Control**
   - - Tissue __________
5. **Final evaluation**
   - - Suitable for HPV analysis
     - Repeat sandwich technique
     - Doubtful
     - Rule out for HPV analysis
6. **External quality control**
   - - Pathologist __________
7. **Internal quality control**
   - - Pathologist __________
8. **Comments** __________
