# Supplementary material for: Human papillomavirus in premalignant oral lesions: No evidence of association in a Spanish cohort
Source: PLoS One. 2019 Jan 16;14(1):e0210070. doi: 10.1371/journal.pone.0210070 (PMC6335078; doi:10.1371/journal.pone.0210070)
Supplement: S1 Table — (DOCX) [file pone.0210070.s002.docx]

**S1 Table. Results from previous studies**

| **Author/Year** | **Country** | **Period of study** | **N Cases** | **HPV+ cases** | **Type of lesion** | **Detection method** |
| --- | --- | --- | --- | --- | --- | --- |
| Llamas-Martinez 2008 [33] | Spain | 2000-2003 | 98 | 45,7% | OL^a^ | PCR^b^ |
| Prigge 2017 [20], [39] | Canada | - | 241 | 6,2% | OLP^c^ | PCR, p16 |
| Arirachakaran 2013 [34] | Thailand | 2008-2010 | 37 | 2,7% | OLP | PCR |
| Khovidhunkit 2008 [35] | Thailand | - | 33 | 0,0% | OLP, OL | PCR |
| Giovannelli 2012 [37] | Italy | - | 99 | 22,0-25,5% | OL, OLP | PCR |
| Rautava 2011 [23] | Finland | Before 1998 | 997 | 23,0-25,4% | OPL^d^, OL | PCR, ISH^e^, DBH^f^ |

^a^OL: oral leukoplakia; ^b^PCR: polymerase chain reaction; ^c^OLP: oral lichen planus; ^d^OPL: oral premalignant lesion; ^e^ISH: in situ hybridization; ^f^DBH: dot blot hibridization
